# Molecular and morphological systematics of a new, reef forming, cupped oyster from the northern Arabian Gulf: *Talonostrea salpinx* new species

**DOI:** 10.3897/zookeys.1043.66992

**Published:** 2021-06-10

**Authors:** Manal Al-Kandari, P. Graham Oliver, Daniele Salvi

**Affiliations:** 1 Ecosystem-Based Management of Marine Resources, Environment and Life Sciences Research Center, Kuwait Institute for Scientific Research, Hamad Al-Mubarak Street, Building 900004, Area 1, Raas Salmiya, Kuwait Kuwait Institute for Scientific Research Kuwait Kuwait; 2 National Museum of Wales, Cathays Park, Cardiff CF10 3NP, UK National Museum of Wales Cardiff United Kingdom; 3 Department of Health, Life & Environmental Sciences - University of L’Aquila,Via Vetoio snc, 67100, L’Aquila-Coppito, Italy University of L’Aquila L'Aquila Italy

**Keywords:** COI, *cox1*, Crassostreinae, crassostreine oyster, DNA sequences, Kuwait, morphology, Ostreidae, 16S rRNA

## Abstract

The rocky northern shores of Kuwait and those of the western, inner shores of Kuwait Bay are dominated by a small, densely encrusting oyster. The identity of this oyster has never been confirmed and was mistaken previously for a small *Saccostrea*. The shell morphology suggests that this species belongs to the subfamily Crassostreinae, but within that subfamily, the presence of marginal erect trumpet-shaped projections is so far unique. Phylogenetic analyses based on mitochondrial DNA sequence data confirmed that this species belongs to the Crassostreinae and has a sister position to the clade including *Talonostrea talonata* and *T. zhanjiangensis*. Genetic distance between this species and *Talonostrea* species is remarkably high, being ~20% for the cytochrome oxidase I gene and ~7% for the 16S rRNA gene. Based on morphological and molecular analyses, this oyster is therefore described here as *Talonostrea salpinx* Oliver, Salvi & Al-Kandari, **sp. nov.** Shell morphology is shown to be variable, and the different forms encountered are described. The wider distribution and origins of this species, whether native or introduced, are discussed.

## Introduction

**Figure 1.**
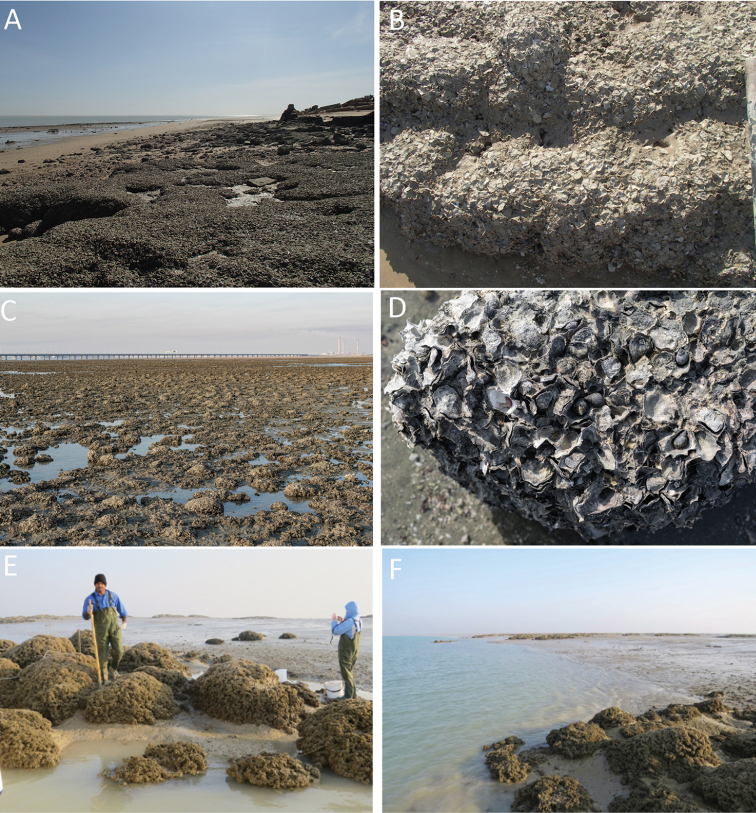
Oyster beds and reefs in northern Kuwait **A, B** Khor Al-Subiyah **C, D** Ashairij **E, F** Boubyan Island (North Khor Al-Subiyah).

The invertebrate fauna of the northern Arabian Gulf and that of Kuwait has a relatively recent investigation period as evidenced by the dearth of specific literature cited by Jones (1986). While Kuwait and the Arabian Gulf were famous for the pearl fishing industry (Al-Shamlãn 2000) and molluscs have been exploited for food for thousands of years (Cataliotti-Valdina 1990; Prieur 2011), the scientific investigation only dates from the 1980s (Al-Bakri et al. 1985). Although famous for pearl oysters (*Pinctada*), true oysters (Ostreidae) are a prominent feature of some shores, particularly around the island of Boubyan (Omar and Roy 2014). The island of Boubyan is listed as a potential world heritage site (https://whc.unesco.org/en/tentativelists/6257/) and is important for breeding and migratory birds such as the Crab Plover. Its waters are home to cetaceans including humpback dolphins, bottlenose dolphins, common dolphins and finless porpoises. Isolated oyster reefs are cited as an important ecological feature (Omar and Roy 2014) around the island of Boubyan where they are known locally as ‘bogar boubyan’ or ‘Boubyan cows’ due to their resemblance to a herd of cattle. These isolated reefs of oysters (Fig. 1E–F) appear at low water where they pose a risk to shipping. These oysters were tentatively identified as *Saccostrea cuccullata* (Born, 1778) in Omar and Roy (2014). The earliest checklist for the Mollusca of Kuwait (Glayzer, Glayzer and Smythe 1984) also lists the dominant oyster at Khor Al-Subiyah (adjacent to Boubyan Island) as *Saccostrea cuccullata*.

Over the years 2014 to 2018, the Kuwait Institute for Scientific Research carried out a survey of the Kuwait’s intertidal fauna and the results for the Mollusca were published in 2020 (Al-Kandari et al. 2020). Two oyster species were common components of the upper and mid intertidal zone. On the eastern coast of Kuwait Bay and the southern coast of the mainland, *Saccostrea* was common and tentatively identified as *S. cuccullata* (Born, 1778). Extensive aggregations of a second, small, oyster were found in Khor Al-Subiyah, adjacent to Boubyan Island, where the upper beach rock is entirely covered by oysters (Fig. 1A, B), extending for many tens of meters. The ‘bogar Boubyan’ mounds were also confirmed to be the same oyster but were not identified as *Saccostrea* but as a species of *Crassostrea*, the lack of marginal chomata confirming the identification. Similar oysters were also found in abundance on the inner Kuwait Bay shores, especially around the peninsula at Ashairij and the Umm Al-Namil Island (Fig. 1C, D).

Glayzer, Glayzer and Smythe (1984) mentions an unidentified *Crassostrea* from the south of Kuwait at An Niggalyat but list the dominant oyster at Khor Al-Subiyah and Ashairij as *Saccostrea cuccullata*. Given that Kathleen Smythe in particular was well acquainted with the Arabian fauna, it is somewhat surprising that she did not recognize the presence of two genera of intertidal oysters. Jones (1986) lists the dominant oyster as *Crassostrea margaritacea* (Lamarck), now *Striostrea margaritacea* (Lamarck, 1819), but he notes that the identification is tentative. From the description given, with shells reaching 100 mm, it seems probable that Jones (1986) was describing *Saccostrea*; *Striostrea* has not been recognized from the northern Gulf. The tentative and incomplete recognition of intertidal oysters of Kuwait by previous authors perhaps illustrates the problematic nature of identifying oysters from their shells alone.

Given that these oysters are key components of the intertidal communities and are cited as a feature for a potential world heritage reserve, their precise identity is important. Consequently, the senior author within Kuwait Institute for Scientific Research (KISR) embarked upon a project to more precisely identify all oyster species in Kuwait based on both morphology and molecular data. Such an integrative taxonomic approach is essential for robust taxonomic identification and systematic assessment of oysters. Indeed, difficulties in identifying and classifying oysters based on a morphological diagnosis extend beyond the species level, up to the genus and subfamily ranks (Salvi et al. 2014; Raith et al. 2016; Salvi and Mariottini 2021) because their morphology is extremely simplified and affected by high levels of phenotypic plasticity (e.g., Liu et al. 2011).

This paper concerns the identity of the oyster listed as *Crassostrea* sp., by Al-Kandari et al. (2020) from Khor Al-Subiyah and Ashairij; future papers will attend to other species including those in the genera *Saccostrea*, *Booneostrea*, *Ostrea* and *Hyotissa*.

## Materials and methods

### Sampling

Representative samples of all shell morphs were collected during the KISR intertidal survey of 2014–2017 (Al-Kandari et al. 2020) and in 2019 further samples were collected specifically for tissue extraction for the molecular study.

**Figure 2.**
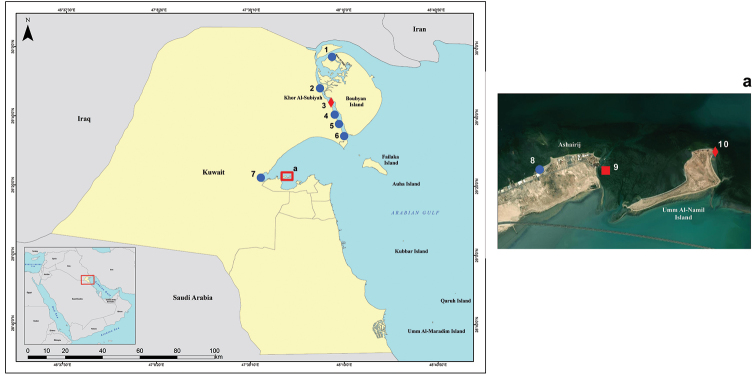
Map of Kuwait indicating known distribution of *Talonostrea salpinx* sp. nov. Blue circle for field records, red diamond for cited material, red square for type locality. Details of localities are given in Table 1.

The sampling sites for the oysters considered here are indicated on the map (Fig. 2) and listed in Table 1. Specimens were levered open and the adductor muscle and mantle were excised whole or in part and fixed in 100% ethanol.

**Table 1.** Sampling transects and localities of the intertidal oysters studied.

Transect	Location Name	Coordinates	Date	References
1	Khor Al-Milh	29.961222, 48.101151	2004–2005	Omar and Roy 2014
2	Boubyan Island (north Khor Al-Subiyah)	29.809521, 48.034599	17.12.2015	Al-Kandari et al. 2020
3	Khor Al-Subiyah (Al Maghasil)	29.74127, 48.09567	23.11.2014	Al-Kandari et al. 2020 and Revisited
4	Khor Al-Subiyah (Al-Alaimah)	29.68225, 48.115972	12.2019	Visited
5	Khor Al-Subiyah (Shumaymah)	29.65672, 48.13083	24.11.2014	Al-Kandari et al. 2020 and Revisited
6	Khor Al-Subiyah (Ras Himar)	29.578667, 48.16803	25.11.2014	Visited
7	Ras Kadmah (Al-Kuwaisat)	29.37795, 47.708	17.11.2014	Al-Kandari et al. 2020
8	Kuwait Bay (Ashairij)	29.38412, 47.83653	03.02.2014	Al-Kandari et al. 2020
9	Between Ashairij and Umm Al-Namil Island	29.383944, 47.849556	29.03.2021	Visited
10	Umm Al-Namil Island	29.38687, 47.87075	29.03.2021	Visited

### Molecular analysis

Total genomic DNA was extracted from 24 alcohol-preserved tissues following standard high-salt protocols (Sambrook et al. 1989). Two mitochondrial gene fragments were amplified by polymerase chain reaction (PCR), the cytochrome oxidase subunit I (*cox1*) and the 16S rRNA (16S). Primers and PCR protocols used for the amplification are described in previous studies (Salvi et al. 2010; Crocetta et al. 2015). Sequencing of PCR products were carried out by the company GENEWIZ (https://www.genewiz.com), using the same primers employed for amplification. Details on sample data and GenBank accession numbers of sequences generated in this study are provided in Table 2 where we also indicated the GenSeq nomenclature for genetic sequences based on the reliability of the taxonomic identification of the source specimens following Chakrabarty et al. (2013). Sequences of both gene fragments were obtained for 22 specimens, whereas for two specimens only 16S sequences were obtained (Table 2). These newly generated sequences were aligned with sequences of 46 oyster species obtained from GenBank and used in a recent phylogenetic assessment of the family Ostreidae (Salvi and Mariottini 2017; see Table 1 of Salvi and Mariottini 2017 for GenBank accession numbers). Multiple sequence alignments were performed with MAFFT v.7 (Katoh and Standley 2013) using the E-INS-i iterative refinement algorithm (alignments available on request from the authors). Genetic distance (uncorrected *p*-distance) between samples analysed in this study and sequences of oyster species obtained from GenBank were calculated with MEGA v.7 (Kumar et al. 2016).

**Table 2.** Genbank accession number, mitochondrial haplotype and GenSeq nomenclature (after Chakrabarty et al. 2013) for genetic sequences obtained from voucher specimens of *Talonostrea salpinx* sp. nov. analysed in this study (na: mitochondrial haplotype not available because the *cox1* sequence was not obtained).

Specimen Catalogue #	Locality	GenBank accession number		Haplotype	GenSeq Nomenclature
		*cox1*	16S		
NMW.Z.2021.009.001 (holotype)	Between Ashairij and Umm Al-Namil Island	MZ126560	MZ099713	Hap1	genseq-1 cox1, 16S
NMW.Z.2021.009.002/1 (paratype)		MZ126561	MZ099714	Hap9	genseq-2 cox1, 16S
NMW.Z.2021.009.002/2 (paratype)		MZ126562	MZ099715	Hap10	genseq-2 cox1, 16S
NMW.Z.2021.009.002/3 (paratype)		MZ126563	MZ099716	Hap1	genseq-2 cox1, 16S
NMW.Z.2021.009.002/4 (paratype)		MZ126564	MZ099717	Hap11	genseq-2 cox1, 16S
NMW.Z.2021.009.002/5 (paratype)		MZ126565	MZ099718	Hap1	genseq-2 cox1, 16S
NMW.Z.2021.009.002/6 (paratype)		MZ126566	MZ099719	Hap12	genseq-2 cox1, 16S
NMW.Z.2021.009.002/7 (paratype)		MZ126567	MZ099720	Hap1	genseq-2 cox1, 16S
NMW.Z.2021.009.002/8 (paratype)		MZ126568	MZ099721	Hap1	genseq-2 cox1, 16S
NMW.Z.2021.009.002/9 (paratype)		–	MZ099722	na	genseq-2 16S
NMW.Z.2021.009.002/10 (paratype)		–	MZ099723	na	genseq-2 16S
NMW.Z.2021.009.002/11 (paratype)		MZ126569	MZ099724	Hap13	genseq-2 cox1, 16S
NMW.Z.2021.009.004/1 (paratype)	Khor Al-Subiyah	MZ126570	MZ099725	Hap1	genseq-2 cox1, 16S
NMW.Z.2021.009.004/2 (paratype)		MZ126571	MZ099726	Hap2	genseq-2 cox1, 16S
NMW.Z.2021.009.004/3 (paratype)		MZ126572	MZ099727	Hap3	genseq-2 cox1, 16S
NMW.Z.2021.009.004/4 (paratype)		MZ126573	MZ099728	Hap1	genseq-2 cox1, 16S
NMW.Z.2021.009.004/5 (paratype)		MZ126574	MZ099729	Hap4	genseq-2 cox1, 16S
NMW.Z.2021.009.004/6 (paratype)		MZ126575	MZ099730	Hap5	genseq-2 cox1, 16S
NMW.Z.2021.009.004/7 (paratype)		MZ126576	MZ099731	Hap1	genseq-2 cox1, 16S
NMW.Z.2021.009.004/8 (paratype)		MZ126577	MZ099732	Hap6	genseq-2 cox1, 16S
NMW.Z.2021.009.004/9 (paratype)		MZ126578	MZ099733	Hap1	genseq-2 cox1, 16S
NMW.Z.2021.009.004/10 (paratype)		MZ126579	MZ099734	Hap7	genseq-2 cox1, 16S
NMW.Z.2021.009.004/11 (paratype)		MZ126580	MZ099735	Hap8	genseq-2 cox1, 16S
NMW.Z.2021.009.004/12 (paratype)		MZ126581	MZ099736	Hap1	genseq-2 cox1, 16S

Phylogenetic relationships were inferred by the Bayesian Inference method in BEAST 2.6.3 (Bouckaert et al. 2019) using the best models of nucleotide substitution selected by JModelTest 2.1.1 (Darriba et al. 2012) under the corrected Bayesian Information Criterion (*cox1*: HKY+G; 16S: GTR+I+G). We unlinked substitution models and clock models of gene partitions, and we linked the tree model across gene partitions. We used the Relaxed Uncorrelated Lognormal Clock model and the Yule process of speciation as tree prior. Two independent runs of 150 million generations were performed, sampling parameters every 15,000 generations. Results were analysed with Tracer 1.7 (Rambaut et al. 2018) to check the runs for convergence (burn-in = 25%). Runs were combined with LogCombiner and a consensus tree representing the posterior distribution was obtained in TreeAnnotator. Nodal support was estimated as Bayesian posterior probability (BPP).

## Results

### Molecular analysis

**Figure 3.**
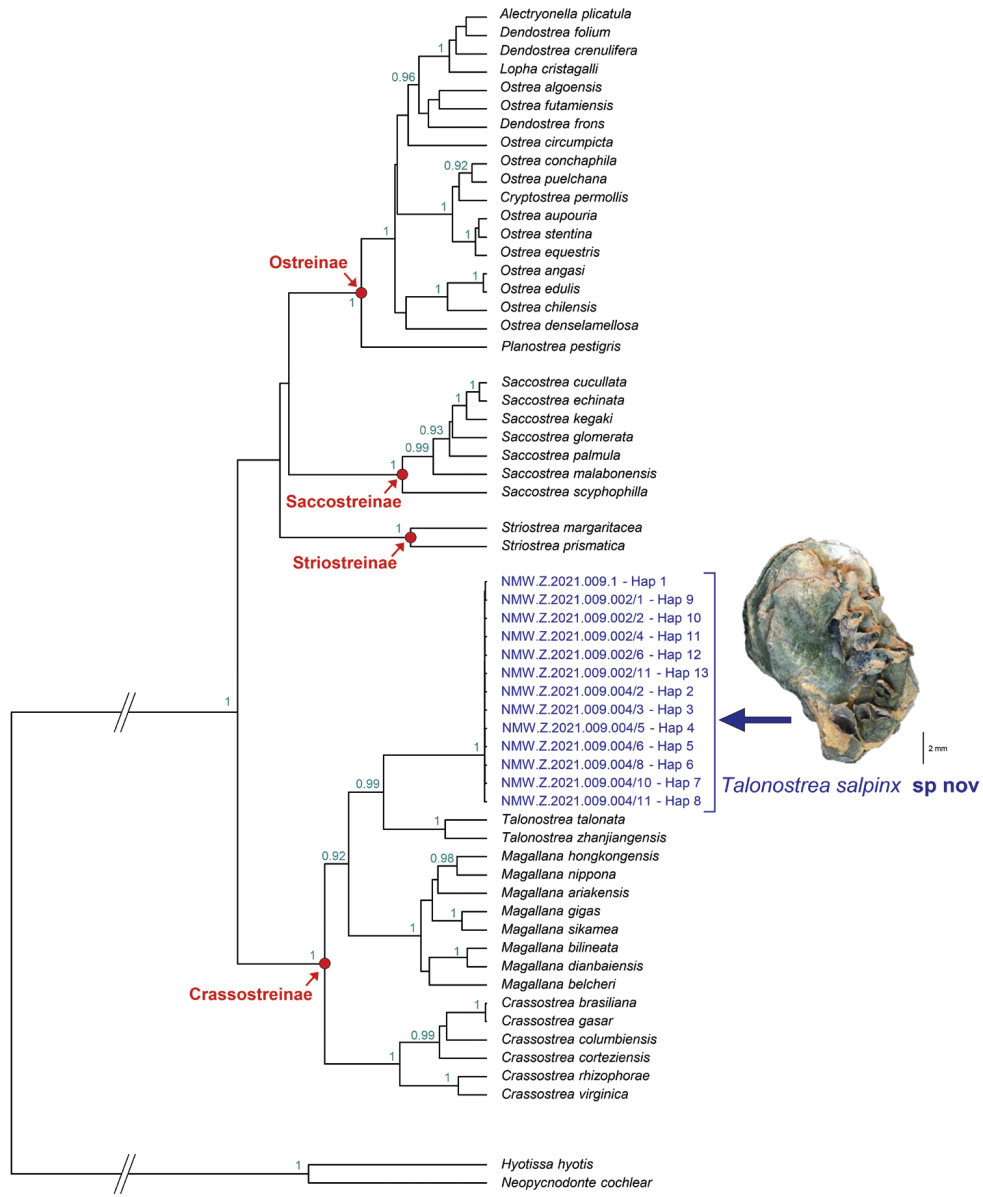
Bayesian phylogenetic tree based on *cox1* and 16S DNA sequence data. Bayesian posterior probability higher than 0.9 are reported in correspondence of the nodes.

Mitochondrial sequences of the oysters from Khor Al-Subiyah and Ashairij represent 13 haplotypes differing from each other by one to three nucleotide substitutions occurring at 17 sites. Given their very limited genetic divergence, all 24 specimens analysed represent a single taxon. Phylogenetic analyses resolve the position of this taxon within the subfamily Crassostreinae as sister to the clade formed by *Talonostrea talonata* and *T. zhanjiangensis* (Fig. 3). This relationship received high statistical support (BPP ≥ 0.99). Genetic distances based on *cox1* between this new taxon and *Talonostrea*, *Magallana* and *Crassostrea* species range from 19 to 20.5%, 17.3 to 20%, and 21.9 to 24.2%, respectively. Genetic distances based on 16S between this new taxon and *Talonostrea*, *Magallana* and *Crassostrea* species range from 6 to 6.9%, 8.1 to 11%, and 16.5 to 21.1%, respectively.

Based on morphological and molecular assessments we assign these oysters to a new *Talonostrea* species that is described in the following section.

### Systematics

**Ostreoidea Rafinesque, 1815**

**Ostreidae Rafinesque, 1815**

**Crassostreinae Scarlato & Starabogatov, 1979**

#### Talonostrea

Taxon classificationAnimaliaOstreidaOstreidae

X.-X. Li & Z.-Y. Qi, 1994

FD41097A-9D72-5660-9487-878B9F227080

##### Type species.

*Talonostrea talonata* X.-X. Li & Z.-Y. Qi, 1994

##### Nominal species included.

*Crassostrea zhanjiangensis* X.-Y. Wu, S. Xiao & Z. Yu, 2013

##### Definition.

The genus *Talonostrea* was first defined on morphological characters alone and was then monotypic, the type species *T. talonata* being described simultaneously by Li and Qi (1994). The oyster took the common name of the ‘cat’s paw oyster’ referring to the folded and broadly digitate margin of the upper valve. This contrasts with *T. zhanjiangensis* and *T. salpinx* (described below), where the upper valve is flat with or without narrow fluted extensions. *Talonostrea salpinx* has the unique feature of possessing trumpet-shaped marginal projections. The shell, therefore, offers few if any defining characters. The anatomical character of a separated style sac observed in *T. talonata* has not been confirmed in *T. zhanjiangensis* and at this time, we cannot be sure if this character is an apomorphy of *T. talonata* or a synapomorphy of the genus as a whole. The anatomical arrangement in *T. salpinx* (see below) agrees with that of *T. talonata* as illustrated in Cavaleiro et al. (2019) and therefore does suggest that this is a defining feature of *Talonostrea*. As the anatomy of *T. zhanjiangensis* has not been described, the genus *Talonostrea* is confirmed on molecular data alone (Salvi and Mariottini 2017) but it is possible that the separate style sac/mid gut character will prove to be a synapomorphy of the genus.

#### Talonostrea salpinx

Taxon classificationAnimaliaOstreidaOstreidae

Oliver, Salvi & Al-Kandari sp. nov.

D489EEA5-3247-55BA-A5B0-93EE7D65EE85

http://zoobank.org/533F31DC-1107-432E-BA79-279793A7C81F

##### Material examined.

**Figure 4.**
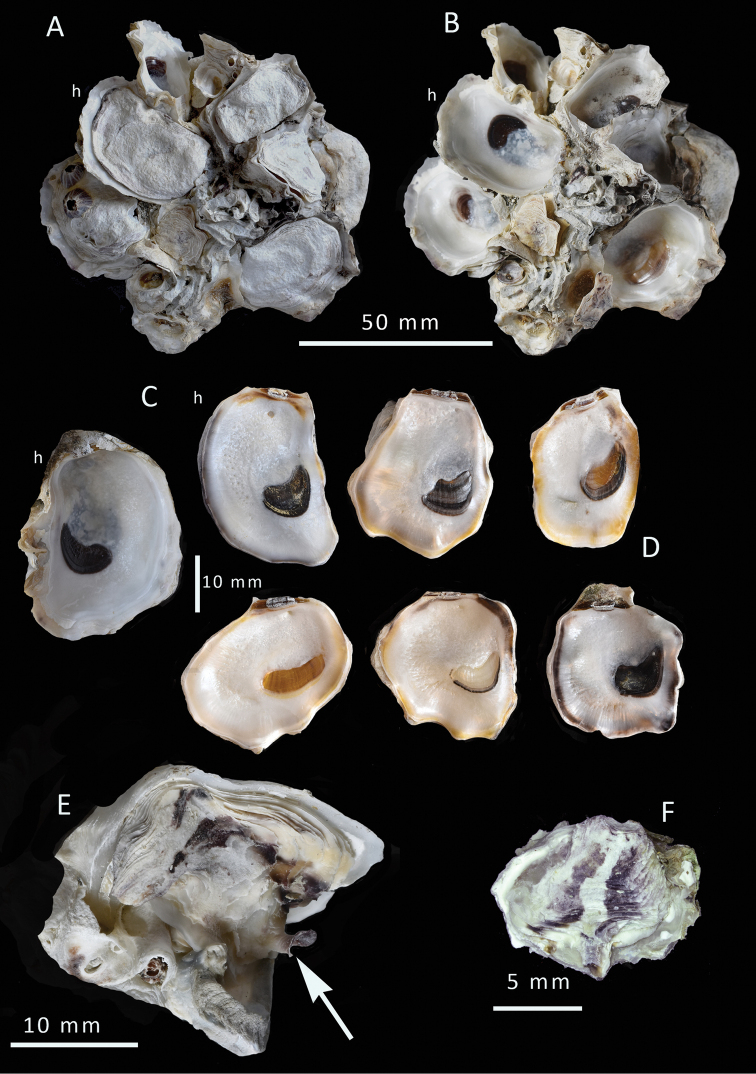
*Talonostrea salpinx* sp. nov. from Ashairij **A, B** clump with and without upper valves, shell h is holotype **C** inner views of lower and upper valves of holotype, NMW.Z. 2021.009. 001 **D** inner views of upper valves of five paratypes showing variation in shape and colouration, NMW.Z.009.002 **E** upper valve with a trumpet shapes projection, arrowed **F** a small upper valve showing radial purple-black colour banding.

All type material deposited in the National Museum of Wales (NMW.Z) Kuwait • 20 + specimens in two clumps; Kuwait Bay, between Ashairij and Umm Al-Namil Island; 29.382423°N, 47.851735°E; intertidal as clumps on rocks and stones; 30 Nov 2019; PG Oliver leg. (Fig. 4). ***Holotype*** (Shell h in Fig. 4A–C) Kuwait • 1 shell; same collection data as for preceding; NMW.Z.2021.009.001; lower valve length 39.1mm, upper valve length 33.1 mm. ***Paratypes*** (Fig. 4D, to illustrate variation in internal colouration only) Kuwait • 11 specimens used in sequencing; collection data as for preceding; NMW.Z. 2021. 009.002/1–11.

##### Other material.

**Figure 5.**
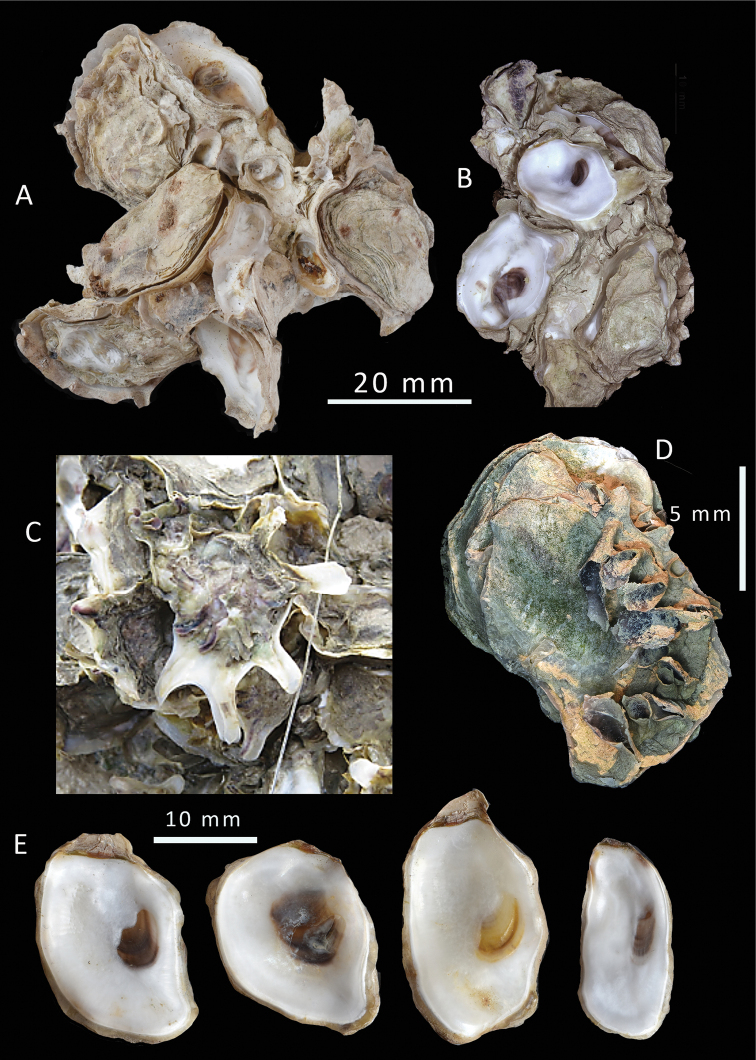
*Talonostrea salpinx* sp. nov. Paratypes from Khor Al-Subiyah, NMW.Z.2021.009.005 **A, B** clump with and without upper valves **C** in situ photograph of a shell from a sheltered position **D** small upper valve with an array of trumpet-shaped projections along margin **E** Inner views of four shells showing variation in shape and internal colouration.

**Figure 6.**
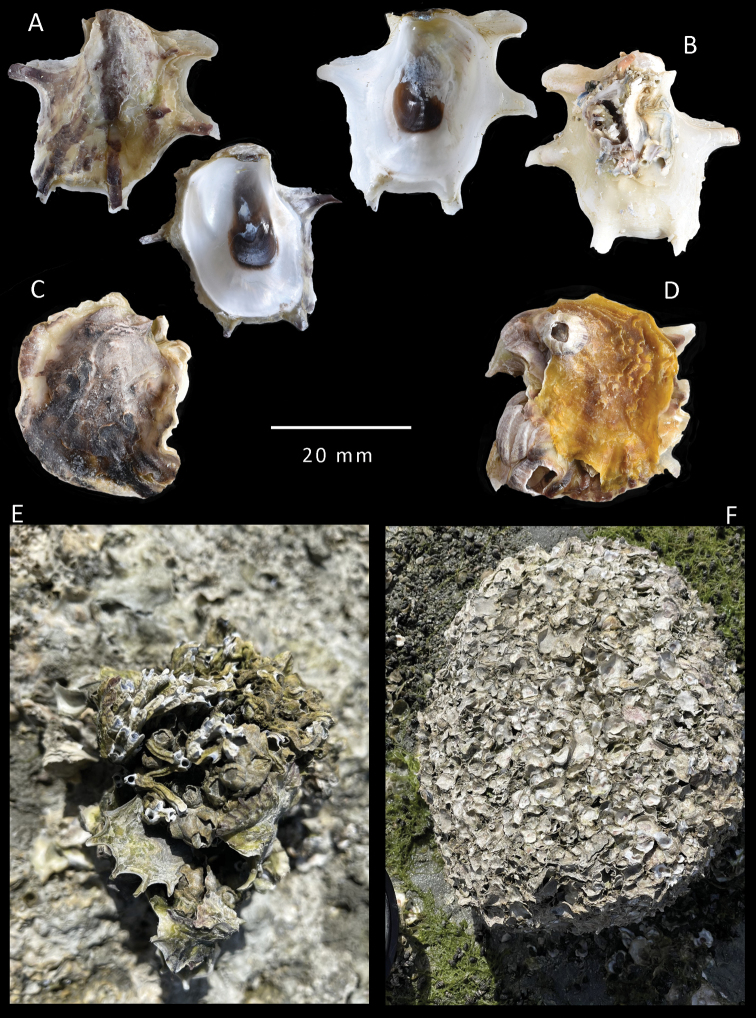
*Talonostrea salpinx* sp. nov. shells from Umm Al-Namil **A, B** external and internal views of a shell with marginal fluted spines, Paratypes NMW.Z.2021.009.006/ **C, D** Paratypes, NMW.Z.2021.009.006/2–3, shells of differing colours and lacking marginal fluted spines **E** clump of shells some with fluted spines associated with the tubeworm *Spirobranchus kraussi* (Baird, 1864) and the barnacle *Amphibalanus amphitrite* (Darwin, 1854). F, rock encrusted with irregular shaped shells mostly lacking fluted spines.

Kuwait • remainder of shells in clumps; same collection data as for preceding; NMW.Z.2021.009.003. Kuwait • 20 + specimens in three clumps; Khor Al-Subiyah, Al Maghasil; 29.74127°N, 48.09567°E; upper intertidal reef forming on beach rock; 15 Nov 2015 and Dec 2019; PG Oliver leg. (Fig. 5). ***Paratypes.*** Kuwait • 12 specimens used in sequencing; collection data as for preceding; NMW.Z.2021.009.004/1–12. ***Paratypes*.** Kuwait • remainder of shells in clumps; same collection data as for preceding, NMW.Z.2021.009.005 (Fig. 5A, B, E). Kuwait • 50 + specimens; Kuwait Bay, Umm Al-Namil island; 29.38687°N, 47.87075°E; on stones, cobbles and rock in the upper intertidal; 29 March 2021; Manal Al-Kandari leg. ***Paratypes*.** Kuwait • 12 specimens; collection data as for preceding; NMW.Z.2021.009.006 (Fig. 6A–C) including shells from dissections.

##### Type locality.

Kuwait, Kuwait Bay, between Ashairij and Umm Al-Namil Island, 29.382423°N, 47.851735°E, intertidal attached to rocks and cobbles, 30 Nov 2019, PG Oliver leg.

##### Derivation of name.

*salpinx*, Greek, a trumpet; referring to the marginal trumpet-shaped projections typical of this species

##### Description.

(**Type series from Ashairij**) Maximum size recorded 41 mm. Specimens of all sizes found growing on or among others creating dense clumps. Shells thin but robust. The lower (left) valve openly cupped, umbonal cavity shallow (Fig. 4B). Margins undulating, slightly raised, roundly digitate and occasionally drawn out into blunt spines. The attachment area is large, furnished with spines and foliations. The inner shell layer is white with brown to black pigmented adductor scars.

Upper (right) valve smaller than and fitting into lower valve (Fig. 4A). Rather flat but undulating, nacreous free margin very narrow. Outline variable, mostly oval some irregularly subquadrate to lingulate. External surface often worn smooth, or weakly foliaceous but not raised into commarginal frills. Occasional shells have open trumpet-shaped projections arising from the margins (Fig. 4E); these are formed by convoluted folding and do not form an entire tube. These trumpet-shaped spines are found mostly in small shells in sheltered sites. Hinge relatively narrow, ligament alivincular, amphidetic; dorsal area not greatly elongated. Chomata absent. External colouration mostly obscured by surface algae but pale grey, some with traces of purple radial streaks, these more prominent in small shells (Fig. 4F). The inner shell layer mostly white, inner margin frequently tinged with pale orange and dark grey, crescentic adductor scar mostly black, some brown, some lacking colour except for a dark ventral rim (Fig. 4C, D)

(**Paratype series from Khor Al-Subiyah**) (Fig. 5A–E) Maximum size recorded 30 mm. Specimens of all sizes forming a continuous reef over beach rock. The shells are thin but not fragile.

The lower valve is deeply cupped often with a deeper umbonal cavity related to the extension of the dorsal hinge plate. Attachment area over most of lower valve with interlocking spines and foliations. Outline is mostly oval but can be distorted into many shapes from lingulate to subcircular; the free margin is upturned, weakly convoluted with short blunt spines; except where growing in sheltered or uncrowded condition where the margins can be greatly extended into spathulate spines (Fig. 5C). Chomata are absent. The ligament is alivincular, the dorsal plate often elongated usually amphidetic but coiling in some. The inner shell layer colour white; adductor scar crescentic reddish-brown to dark brown/black in colour, colouration often extending into the umbonal cavity.

Upper valve smaller than, and fitting into lower valve. Rather flat but undulating, nacreous free margin very narrow slightly elevated. External surface weakly foliaceous but not raised into commarginal frills. Shells sheltered among others and juveniles frequently display open trumpet-shaped projections as above (Fig. 5D). External colouration is mostly obscured by algal growth but is underlying greyish-beige; juvenile shells and those in sheltered positions may have coloured radial bands of a purple-black hue (Fig. 5C). Chomata are absent. The inner shell layer is white with the crescentic adductor scar brown to brown-black in colour (Fig. 5E).

(**Paratype series from Umm Al-Namil**) (Fig. 6A–D) Maximum size recorded 35 mm. Specimens of all sizes are found attached in clumps (Fig. 6E) to stones and cobbles or encrusting rocks (Fig. 6F). While most of these are identical to the type series (Fig. 6F) those from sheltered sites are rather thin, may have marginal extensions that are easily broken and often exhibit a more vivid colouration.

Attachment area small, free area with 5–7 prominent folds extending as furrowed spines (Fig. 6B). Outline is mostly oval but can be distorted into many shapes from lingulate to subcircular. Chomata absent. The ligament alivincular, dorsal plate often elongated usually amphidetic but coiling in some. The inner shell layer colour white: adductor scar crescentic reddish-brown to dark brown/black in colour, colouration often extending into the umbonal cavity (Fig. 6B).

Upper valve smaller than, and fitting into lower valve. Rather flat but undulating, nacreous free margin narrow, slightly elevated and extended as spines (Fig. 6A) fitting into furrows of lower valve. External surface weakly foliaceous, not raised into commarginal frills. External colouration ranging from uniformly dull grey, dirty white, to beige with purple-brown radial stripes extending onto spines (Fig. 6A); few golden brown (Fig. 6D) to purple-black all over (Fig. 6C). Chomata absent. Nacreous layer white with the crescentic adductor scar brown to brown-black in colour (Fig. 6A).

**Figure 7.**
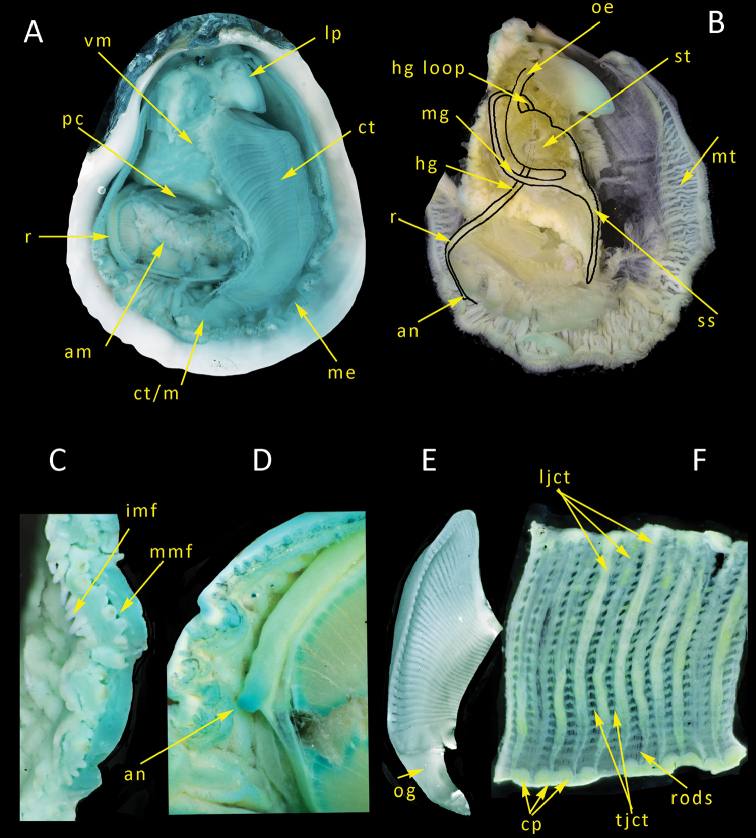
Anatomy of *Talonostrea salpinx* sp. nov. **A** gross view after removal of upper (right) valve **B** gross view including route of alimentary canal after removal of ctenidia, gonad and digestive diverticula **C** mantle edge **D** rectum and anus **E** excised labial palp **F** portion of ctenidium showing fine structures. Abbreviation: am, adductor muscle; an, anus; cp, ciliated pad; ct, ctenidium; ct/m, ctenidium mantle edge junction; hg, hind gut; hg loop, hind gut loop behind stomach; imf, inner mantle fold; ljct, longitudinal junction; lp, labial palp; me, mantle edge; mg, mid gut; mmf, middle mantle fold; mt, mantle; oe,oesophagus; og, oral groove; pc, pericardium; r, rectum; rods, ctenidial filaments; s, stomach; ss, style sac; tjct, transverse junction; vm, visceral mass.

***Anatomy*** (Fig. 7). Preserved specimens from Umm Al-Namil were opened by severing the ligament, levering the upper valve open slightly and then slicing the adductor muscle to free the upper valve. The animals were then dissected by sequentially removing the mantle (Fig. 7A), the ctenidia and finally dissecting into the visceral mass removing gonad and digestive diverticula tissue to reveal the alimentary system (Fig. 7B). Tissues have been stained in Methyl Blue to aid contrast.

The mantle in its preserved and contracted condition shows an array of radial folds (Fig. 7B). Mantle edge free except at the ventral margin where it is joined to the ctenidium. Mantle edge with three folds, middle fold with short pigmented, tuberculated, tentacles typically arranged with a one large one small pattern (Fig. 7C), inner fold with simple smooth unpigmented tentacles all of equal size and shape (Fig. 7C).

Adductor muscle crescent shaped in a posterior ventral position; pericardium immediately dorsal to it (Fig. 7A). Ctenidium of two reflected demibranchs (Fig. 7A), filamental rods bundled into groups of 10–12 by longitudinal and transverse junctions (Fig. 7F). Labial palps triangular, inner faces entirely with sorting ridges, oral groove smooth, short (Fig. 7E).

Alimentary system (Fig. 7B) of large stomach within visceral mass dorsal of pericardium, surrounded by digestive diverticula; oesophagus enters dorsally; mid gut and style sac open on lower anterior side of visceral mass; style sac long, curving ventrally towards adductor muscle; mid gut running towards the posterior below the stomach and then hind gut travels on the posterior side dorsally before plunging under the stomach, curving ventrally, appearing through the pericardium and running as rectum around posterior of adductor muscle; anus simple slightly elevated (Fig. 7D).

##### Habitat.

*Talonostrea salpinx* is an oyster of the upper and middle shores living attached to exposed hard substrates. Extensive oyster growth is seen in Khor Al-Subiyah and the western end of Kuwait Bay. The waters of these localities are highly turbid and often hypersaline (Al-Yamani et al. 2004), the intertidal environment is further stressed by experiencing a summer air temperature maximum of 50°C and a winter minimum occasionally as low as 0°C. The summer salinity at Ashairij has been measured at 47 ppt whereas to the south it is around 45 ppt (Pokavinich et al. 2013). In Khor Al-Subiyah the salinity can be variable depending on the discharge from the Tigris and Euphrates rivers through the Shatt el Arab (Omar and Roy 2014). The indications are that *T. salpinx* can survive multiple extremes of turbidity, air temperature and salinity.

##### Distribution.

*Talonostrea salpinx* has been found or recorded from a number of locations other than that cited in Material examined. The current distribution can be summarised as the south-eastern area of Kuwait Bay, from Raz Kazmah to Umm Al-Namil Island where extensive fields are present, and the oysters are attached to low rocks and loose cobbles. Throughout Khor Al-Subiyah, including Khor Al-Milh adjacent to Warbah Island in the very north of Boubyan, where oysters form intertidal reefs and mounds. It has also been found at an unlisted locality in Iran (see Discussion).

##### Remarks.

The shell morphology of *T. salpinx* is in keeping with other crassostreines in lacking any chomata. Unusual for the subfamily is the presence of the trumpet-shaped marginal projections as these are not recorded for any other Indo-Pacific *Magallana* or *Talonostrea* nor indeed for any Atlantic *Crassostrea* (Inaba and Torigoe 2004). The adductor scar is strongly pigmented in the larger shells, a character not shared by *T. talonata* but present in *T. zhanjiangensis*.

*Talonostrea talonata* is known as the ‘cat’s paw oyster’ (Li and Qi 1994; Cavaleiro et al. 2019) due to it having a strongly ridged and digitate upper valve and in this feature is very different from *T. salpinx*. The only other *Talonostrea* is *T. zhanjiangensis*Wu et al. 2013. and here there is greater similarity with *T. salpinx* in having a weakly undulating cupped lower valve and a rather flat featureless upper valve but lacking trumpet-shaped marginal projections. Due to the more rounded upper valve, *T. zhanjiangensis* has been given the common name of the “cats ear oyster” (Wu et al. 2013), perhaps *T. salpinx* should be known as the ‘tufted cat’s ear oyster’ in reference to the marginal projections.

The morphology and molecular results of *T. salpinx* clearly indicate that this new species belongs to the Pacific cupped oyster lineage, with a closer affinity to the Chinese species of *Talonostrea* rather than to the more widespread *Magallana* species. This is supported also in the morphology where both *T. salpinx* and *T. talonata* share the character of the style sac and mid gut being separate for most of their lengths while in *Magallana* and *Crassostrea* the mid gut and style sac run together. A discrepancy between the route of the mid gut as illustrated by Li and Qi (1994) and that of Cavaleiro et al. (2019) for *T. talonata* exists. In Li and Qi (1994) the mid gut of *T. talonata* is shown running to the anterior before curving over the face of the stomach whereas in Cavaleiro et al. (2019), and in *T. salpinx*, the mid gut runs toward the posterior. Without Chinese specimens to dissect we are unable to tell if the difference is real or an artifact in the illustration by Li and Qi (1994). The detailed anatomy of *T. zhanjiangensis* has never been described. Torigoe (1981) described the mantle tentacles of the major genera noting that for the inner fold of Crassostreinae the tentacles are arranged in an alternating large-small pattern but in *T. salpinx* the inner mantle fold tentacles are all of the same size. The mantle tentacle arrangement has not been described for other *Talonostrea* species. Genetic distance between *T. salpinx* and the other two *Talonostrea* species is remarkably high (~20% and ~7% for *cox1* and 16S genes respectively). Such a high genetic divergence combined with a unique morphology might justify the assignment of this new species to a distinct genus of Crassostreinae. However, we believe that its assignment to the genus *Talonostrea* is a more conservative approach as it avoids erecting a monotypic genus. This study emphasizes once again that our knowledge of the evolutionary diversity of oysters is far from complete and that molecular data are essential for a robust taxonomic identification and classification of oyster taxa.

## Discussion

It is perhaps surprising that the Kuwait oyster belongs to the genus *Talonostrea* as that genus has its distribution centred on China rather than to the more widespread *Magallana*. Some northern Gulf bivalves, such as *Congetia chesnyi* (Oliver & Chesney, 1994) and *Protapes cor* (Sowerby, 1853) are not found further south in the Gulf but occur again in Pakistan and northern India. One might have expected the Kuwait oyster to be allied to species such as *Magallana bilineata* (Röding, 1798) or *M. cuttackensis* (Newton & Smith, 1912), both widely recorded from the west coast of India and Pakistan, and the former also found as a non-native in eastern Australia (Willan et al. 2021). Comparison of 16S rRNA sequences generated in this study for *T. salpinx* with sequences available from GenBank indicate that the same taxon has been found in Iran but was not identified (GenBank accession numbers HF549037–HF549058). Sequence identity between oysters from Kuwait and oysters from Iran is between 99.3 and 100%. Currently *T. salpinx* is restricted to the northern Arabian Gulf and is perhaps endemic to this region. The lack of presence records for *T. salpinx* outside the northern Gulf might be explained by the fact that this species has been overlooked (or become extinct) from a wider range or that it originated *in situ*. This latter hypothesis requires a rapid rate of speciation as the Arabian Gulf was dry at the last glacial maximum some 18, 000 years ago with the present shoreline reached some 6000 years ago (Lambeck 1996). Based on the observed phylogenetic divergence of *T. salpinx* with *T. talonata* or *T. zhanjiangensis*, their separation is likely to be several million years old as deduced by comparison of branch lengths within Crassostreinae as estimated in this study and in previous phylogenetic analyses implementing molecular clock models (the estimated divergence between *Magallana* and *Crassostrea* is of 66–102 Mya according to Ren et al. 2010). However, it is not possible to exclude the fact that an unknown sister species of *T. salpinx* occurs elsewhere.

**Figure 8.**
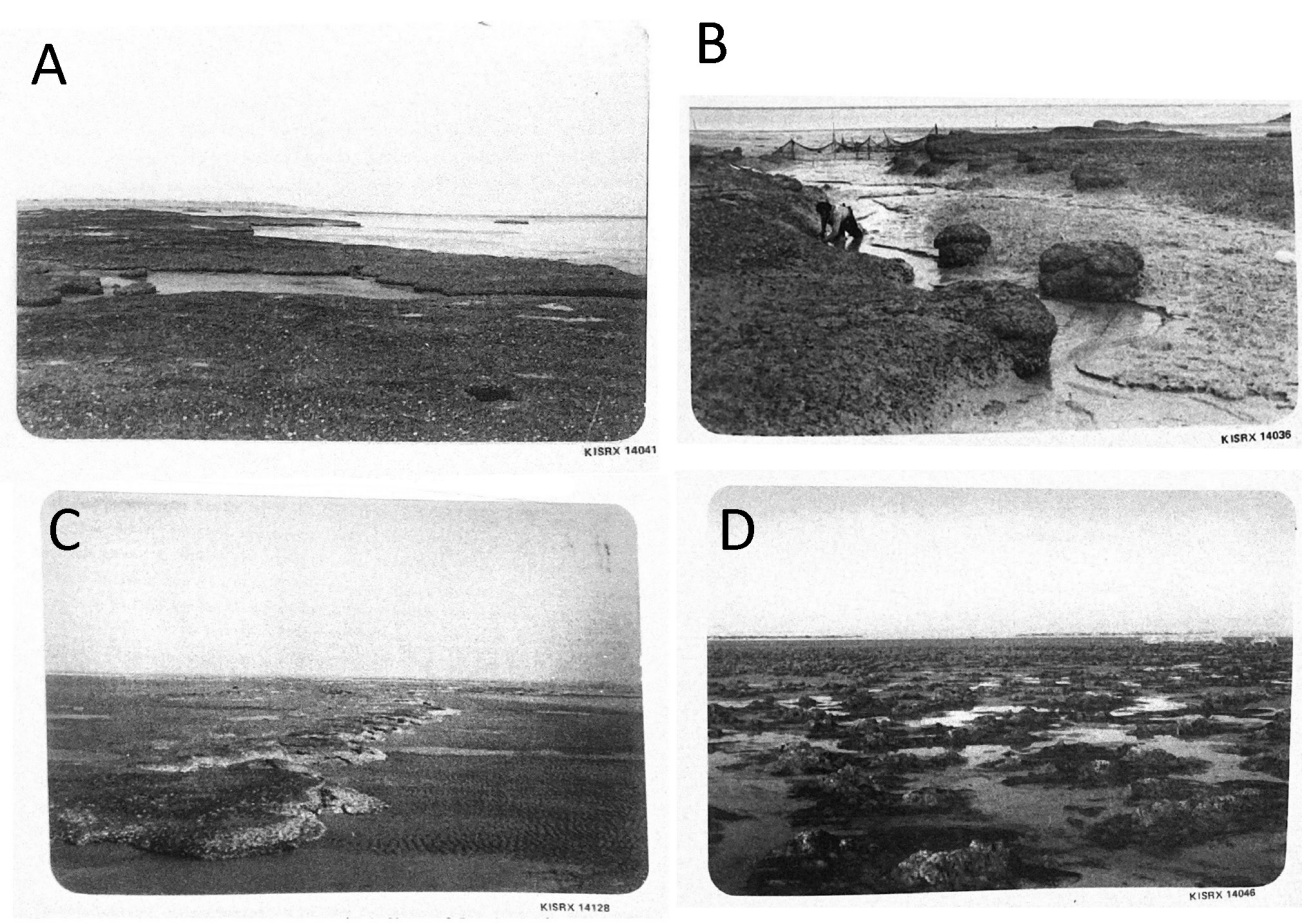
Photographs of *Talonostrea salpinx* sp. nov. beds and reefs from Al-Bakri et al. 1985**A–C** reefs and beds in Khor Al-Subiyah **D** oyster field at Al-Memlahah, south-eastern end of Kuwait Bay.

*Talonostrea talonata* has now been recognised in Peru, Brazil and Argentina, indicating that *Talonostrea* can be invasive (Cavaleiro et al. 2019). If *T. salpinx* is alien in the Arabian Gulf, then its origins are unknown and at this time we have little data on the age of the oyster reef at Khor Al-Subiyah or the oyster field at Ashairij. Al-Bakri et al. (1985) includes photographs of the oyster reefs in Khor Al-Subiyah and the oyster field at Ashairij and these illustrate how extensive and well developed the reefs were at that time (Fig. 8). Given that the oyster mounds had been given the colloquial name of bogar Boubyan (cows) one would surmise that they were a long-time feature of the landscape. Marine invertebrate invasions are often cited as a result of transport of larvae in ballast water (Gonçalves 2013) but in Kuwait relatively few (fourteen) invasive organisms have been recognised (Al-Yamani, Skryabin and Subba Rao 2015). These latter authors cite the extreme environment of the northern Gulf preventing colonization by many species of fauna and flora and this might suggest that *T. salpinx* is not an invasive species. However, it is apparent that *T. salpinx* thrives in these conditions and is well adapted to great variations in water quality suggesting to us that it is a native species.

## Supplementary Material

XML Treatment for Talonostrea

XML Treatment for Talonostrea salpinx
